# Commentary: The plasmacytoid dendritic cell paradox in cancer: impaired type I interferon responses in a nucleic acid–rich tumor microenvironment

**DOI:** 10.3389/fimmu.2026.1941483

**Published:** 2026-09-23

**Authors:** Jin Dou Yao, Jiao Yue Shuang

**Affiliations:** 1 Department of Ultrasound Medicine, Suining Central Hospital, Suining, China; 2 Department of Medical Imaging, Suining Central Hospital, Suining, China

**Keywords:** cancer immunotherapy, nucleic-acid delivery, plasmacytoid dendritic cells, TLR7, TLR9, tumor microenvironment, type I interferon

## Introduction

Vasilakou et al. provide a timely explanation for the paradox of abundant tumor-derived nucleic acids coexisting with weak type I interferon (IFN-I) production by tumor-associated plasmacytoid dendritic cells (pDCs) (1). Their focus on ligand access to endosomal Toll-like receptors (TLR)7 and TLR9 is important: extracellular nucleic-acid abundance does not establish productive receptor engagement. Our conceptual advance is a causal-discrimination framework (**Figure 1**), rather than a claim that internalization is invariably rate-limiting. It distinguishes among ligand availability, uptake, endosomal trafficking, receptor-proximal signaling, cellular state/identity, and microenvironmental inhibition. This decomposition identifies experimentally separable explanations for a low IFN-I response and extends the access-centered interpretation offered by Vasilakou et al.

**Figure 1:**
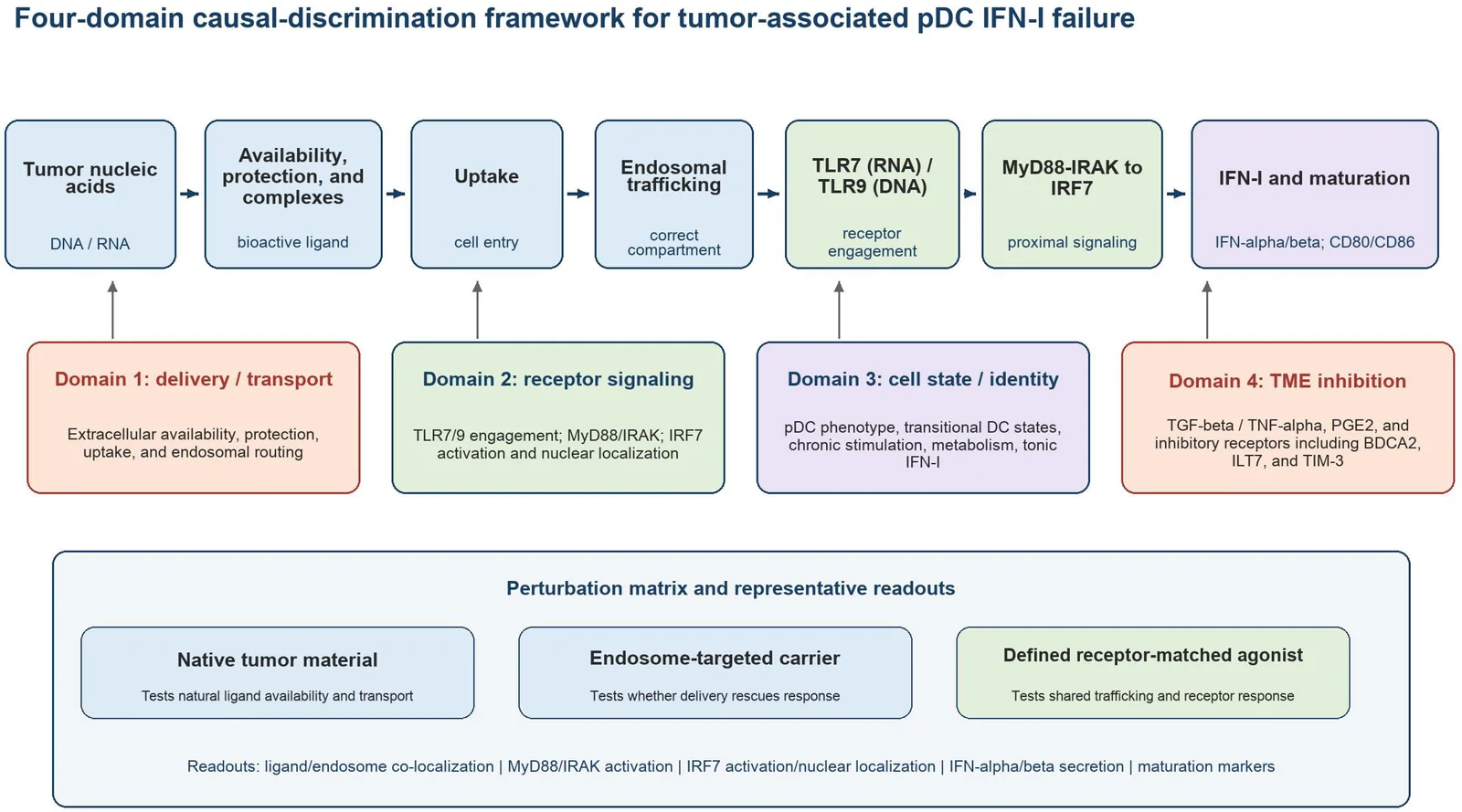
Four-domain causal-discrimination framework (Image 1) for low pDC IFN-I output in tumors. Tumor nucleic acids must progress through availability/protection, uptake, endosomal trafficking, TLR7 or TLR9 engagement, and MyD88-IRAK-IRF7 signaling to induce IFN-I and maturation. Cell state/identity and TME-mediated inhibitory signals modify this pathway. Comparing native tumor material, endosome-targeted delivery, and defined receptor-matched agonists with viability, uptake, localization, proximal signaling, and distal functional controls can narrow, but not by itself prove, the dominant defect.

## From nucleic-acid abundance to receptor engagement

TLR9 and TLR7 should not be treated as interchangeable. TLR9 recognizes DNA and its endosomal restriction is a safeguard against inappropriate self-DNA sensing (2); TLR7 recognizes RNA and has distinct ligand and trafficking biology. Thus, tumor DNA and RNA should be measured and perturbed separately. LL-37 can protect and deliver otherwise weakly stimulatory self-DNA to pDC endosomes (3), establishing a delivery principle without demonstrating that equivalent complexes form or persist in tumors. Likewise, STING-dependent sensing of tumor DNA by conventional dendritic cells (4) does not demonstrate access to pDC endosomes. Tumor nucleic acids must therefore be assessed for extracellular availability, protection/complex formation, cellular uptake, and delivery to the appropriate endosomal compartment.

## A four-domain framework for low pDC IFN-I output

We propose four interacting domains: (i) delivery and transport, encompassing ligand availability, uptake, and endosomal routing; (ii) receptor-proximal signaling, including TLR7/9 engagement, myeloid differentiation primary response 88‌ (MyD88)/interleukin-1 receptor-associated kinase (IRAK) signaling, and interferon regulatory factor 7 (IRF7) activation; (iii) cell state and identity, encompassing pDC heterogeneity, differentiation, chronic stimulation, metabolism, and prior IFN-I exposure; and (iv) tumor microenvironment (TME)-mediated inhibition. The latter includes transforming growth factor-beta (TGF-beta), TNF-alpha, and prostaglandin E2 (PGE2), as well as tumor-associated ligands engaging inhibitory receptors such as blood dendritic cell antigen 2 (BDCA2), ILT7, and TIM-3, which can directly restrain pDC activation and IFN-I production (5, 9).

Cell identity is integral to this framework. pDC-enriched gates may contain related or transitional dendritic-cell states, and intersectional mouse models show the limitations of relying on a single marker (7). For flow-cytometry studies, a practical starting strategy is to identify viable singlets, lineage-negative (CD3, CD14, CD16, CD19, CD20, CD56, and CD335), HLA-DR-positive, CD123-high, CD303/BDCA2-positive, and CD304/BDCA4-positive cells, with CD11c, AXL, and SIGLEC6 recorded to identify or exclude related states. This panel should be adapted to tissue and dissociation context and paired with a prespecified functional readout, rather than interpreting pDC abundance alone as a biomarker (9, 13). Chronic TLR stimulation sustains exhausted pDC states in viral infection (6), but this finding requires direct validation in cancer. Recent mouse data also indicate that tonic IFN-I signaling can shape pDC heterogeneity and subsequent responsiveness; this is a contextual-state mechanism, not yet direct evidence for human tumor-associated pDCs (11).

## An experimentally testable perturbation matrix

A paired matrix can narrow the dominant defect. Compare native tumor nucleic acids or tumor interstitial material, the same material in an endosome-targeted carrier, and defined synthetic agonists matched to TLR7 or TLR9. These conditions do not form a simple upstream-versus-downstream test: cytosine-phosphate-guanine; TLR9: Toll-like receptor 9 (CpG/TLR9) agonists also require uptake and appropriate endosomal trafficking (14). Predefine cell viability and recovery, ligand/endosome colocalization, receptor abundance and agonist activity, MyD88/IRAK activation, IRF7 activation or nuclear localization, IFN-alpha/beta secretion, and maturation markers. Rescue by targeted delivery with preserved responses to a defined agonist would support inefficient availability, complexing, or cargo-specific transport. Failure across conditions, after these controls, would implicate a shared trafficking, receptor-proximal, state-dependent, or TME-mediated mechanism; it should not automatically be labeled intrinsic pDC dysfunction without direct evidence.

## Therapeutic implications and limits

This framework refines, rather than replaces, existing TLR-directed approaches. TLR agonists have extensive preclinical and clinical development, including intratumoral CpG-A TLR9 agonist vidutolimod in combination with PD-1 blockade (10, 12). These experiences support mechanism-matched testing, but do not establish that a TLR agonist will overcome downstream inhibition in every tumor. Conversely, the observation that IFN-lambda can prime pDC TLR7 responses while countering TGF-beta/PGE2-mediated suppression is an emerging therapeutic hypothesis (8). Its relevance to human tumor-associated pDCs and its anticancer efficacy remain to be determined.

## Discussion

The internalization/access hypothesis is valuable because it is testable, not because it supplies a universal hierarchy. By distinguishing delivery/transport, receptor signaling, cell state/identity, and TME-mediated inhibition, the proposed framework can guide experiments toward the level at which a response fails. It may provide a framework for future mechanism-based stratification, while avoiding premature clinical claims and the conflation of ligand-access failure with experimentally demonstrated pDC-intrinsic dysfunction.
